# Supplementary material for: Demographic Control Measure Implications of Tuberculosis Infection for Migrant Workers across Taiwan Regions
Source: Int J Environ Res Public Health. 2022 Aug 11;19(16):9899. doi: 10.3390/ijerph19169899 (PMC9408672; doi:10.3390/ijerph19169899)
Supplement: Supplementary file 1 [file ijerph-19-09899-s001.zip › ijerph-1812133-supplementary.pdf]

Supplementary material: Tables S1 and S2; Figures S1-S3.

Table S1 Distribution of migrant workers in different administrative regions in Taiwan

**Administrative regions**	**Numbers of migrant workers**	**Proportion [a]**	**Indonesia (N, %) [b]**	**Philippines (N, %)**	**Thailand (N, %)**	**Vietnam (N, %)**
Taoyuan City	91,898	4.14%	11,935, 13%	30,705, 33%	16,998, 19%	32,260, 35%
Taichung City	78,423	2.80%	12,031, 15%	15,690, 20%	10,215, 13%	40,487, 52%
New Taipei City	56,041	1.40%	8,853, 16%	9,012, 16%	7,753, 14%	30,422, 54%
Tainan City	44,240	2.35%	6,128, 14%	13,945, 32%	5,335, 12%	18,832, 43%
Changhua County	43,969	3.44%	8,936, 20%	5,351, 12%	6,086, 14%	23,596, 54%
Kaohsiung City	38,815	1.40%	6,771, 17%	13,508, 35%	2,986, 8%	15,550, 40%
Hsinchu County	22,126	3.97%	1,246, 6%	13,016, 59%	1,983, 9%	5,881, 27%
Miaoli County	13,573	2.47%	1,854, 14%	5,709, 42%	1,730, 13%	4,280, 32%
Yunlin County	11,170	1.63%	1,901, 17%	1,456, 13%	1,546, 14%	6,267, 56%
Hsinchu City	10,488	2.35%	512, 5%	8,036, 77%	241, 2%	1,699, 16%
Pingtung County	7,533	0.91%	2,641, 35%	1,116, 15%	419, 6%	3,357, 45%
Yilan County	6,875	1.51%	2,101, 31%	1,603, 23%	603, 9%	2,568, 37%
Nantou County	6,722	1.35%	1,187, 18%	787, 12%	1,562, 23%	3,186, 47%
Chiayi County	6,545	1.29%	1,254, 19%	1,317, 20%	1,041, 16%	2,933, 45%
Taipei City	2,574	0.10%	501, 19%	135, 5%	785, 31%	1,153, 45%
Penghu County	2,307	2.21%	1,921, 83%	258, 11%	0, 0%	128, 6%
Keelung City	2,104	0.57%	692, 33%	429, 20%	240, 11%	743, 35%
Hualien County	1,869	0.57%	438, 23%	463, 25%	617, 33%	351, 19%
Chiayi City	732	0.27%	214, 29%	134, 18%	44, 6%	340, 46%
Taitung County	472	0.22%	271, 57%	32, 7%	0, 0%	169, 36%
Kinmen County	217	0.16%	32, 15%	0, 0%	101, 47%	84, 39%
Lienchiang County	60	0.46%	27, 45%	11, 18%	0, 0%	22, 37%

[a] The proportion is defined as the number of migrant workers to total population size (migrant add local population) in specific administrative regions.
[b] N and % indicated population size and percentage in specific administrative regions.

Table S2 Percentage of reduction in total number of latently infected (L) + infectious (T) individuals under different single, dual, and triple combinations control strategies in Taoyuan, Taichung, and New Taipei Cities in 2020 (two years projection) and 2023 (five years projection)

**Projection**	**City**	**Single control strategies**								
		Social distance control (u_1)								
		10%	20%	30%	40%	50%	60%	70%	80%	90%
2-yr	Taoyuan City	5.8%	11.3%	16.4%	21.3%	25.9%	30.3%	34.4%	38.3%	42.1%
	Taichung City	7.3%	14.1%	20.4%	26.3%	31.9%	37.0%	41.9%	46.4%	50.7%
	New Taipei City	10.9%	20.7%	29.5%	37.5%	44.7%	51.2%	57.2%	62.6%	67.6%
5-yr	Taoyuan City	18.1%	31.8%	42.4%	51.0%	58.1%	64.0%	68.9%	73.1%	76.7%
	Taichung City	24.2%	40.5%	52.2%	61.0%	68.0%	73.5%	78.0%	81.6%	84.7%
	New Taipei City	52.7%	72.2%	81.4%	86.8%	90.4%	92.8%	94.6%	96.0%	97.0%
		Early screening control (u_2)								
		10%	20%	30%	40%	50%	60%	70%	80%	90%
2-yr	Taoyuan City	3.8%	7.6%	11.3%	14.9%	18.4%	21.8%	25.2%	28.5%	31.7%
	Taichung City	4.6%	9.1%	13.5%	17.7%	21.8%	25.9%	29.7%	33.5%	37.2%
	New Taipei City	6.2%	12.1%	17.8%	23.3%	28.5%	33.5%	38.2%	42.8%	47.1%
5-yr	Taoyuan City	12.2%	22.9%	32.4%	40.8%	48.2%	54.8%	60.6%	65.8%	70.5%
	Taichung City	16.2%	29.5%	40.7%	50.0%	57.9%	64.6%	70.2%	75.0%	79.2%
	New Taipei City	32.5%	54.9%	68.7%	77.4%	83.4%	87.6%	90.6%	92.9%	94.7%
		DOTS control (u_3)								
		80%	85%	90%	95%					
2-yr	Taoyuan City	13.3%	13.9%	14.5%	15.1%					
	Taichung City	16.7%	17.5%	18.2%	18.9%					
	New Taipei City	22.7%	23.8%	24.8%	25.7%					
5-yr	Taoyuan City	40.9%	42.3%	43.5%	44.7%					
	Taichung City	50.3%	51.7%	53.0%	54.3%					
	New Taipei City	77.3%	78.4%	79.5%	80.5%					

		**Dual and triple combinations control strategies**			
		u_1 (70%)+u_2 (70%)	u_1 (70%)+u_3 (85%)	u_2 (70%)+u_3 (85%)	u_1 (70%)+ u_2 (70%)+u_3 (85%)
2-yr	Taoyuan City	39%	38%	31%	41%
	Taichung City	47%	46%	37%	49%
	New Taipei City	63%	62%	49%	66%
5-yr	Taoyuan City	74%	73%	68%	76%
	Taichung City	83%	82%	77%	84%
	New Taipei City	96%	96%	94%	97%

Figure S1 Sensitivity Analysis of four transmission rates (β_M, β_L, β_{LM}, β_{ML}) and reactivation rate (k) in Taoyuan City.

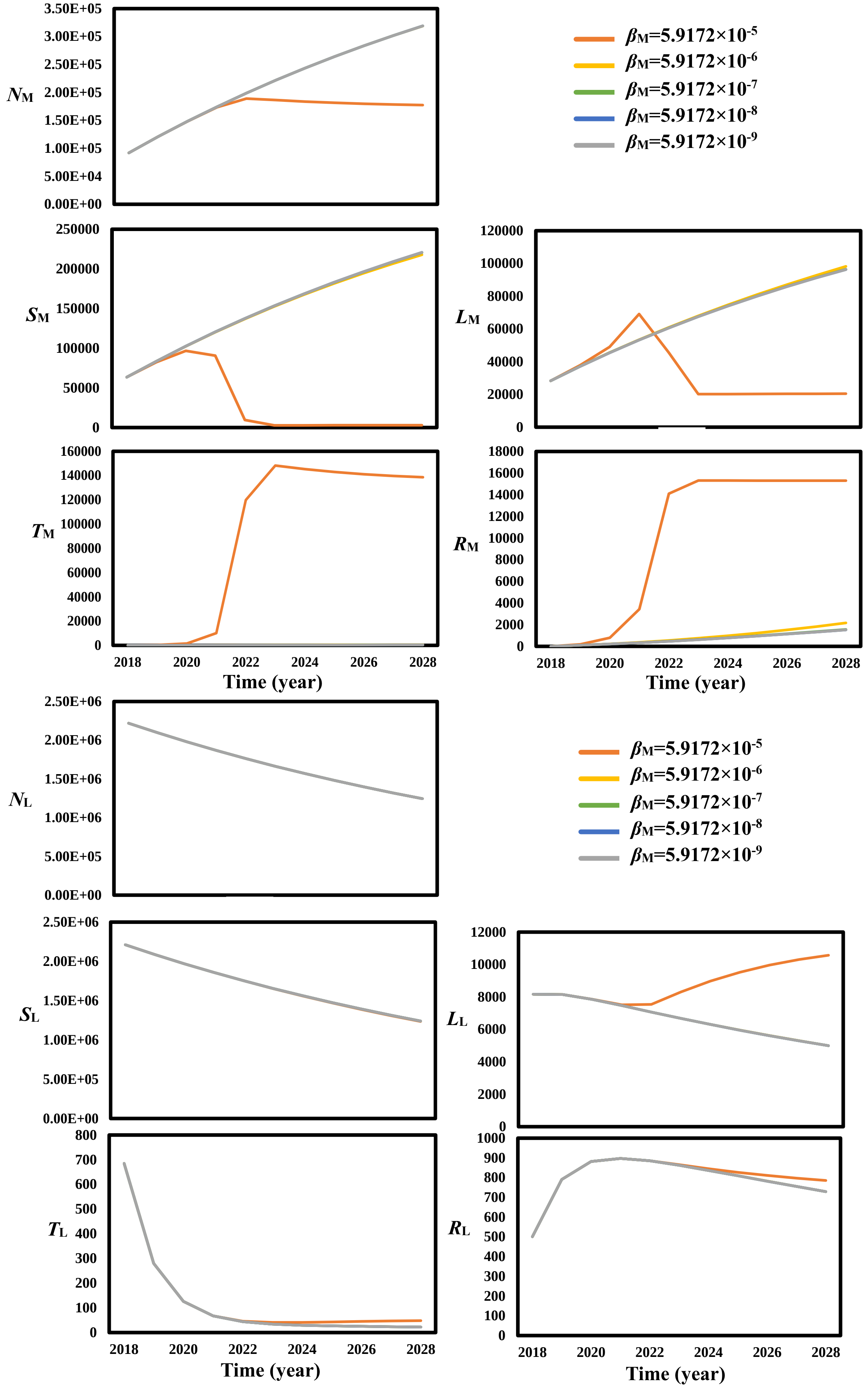

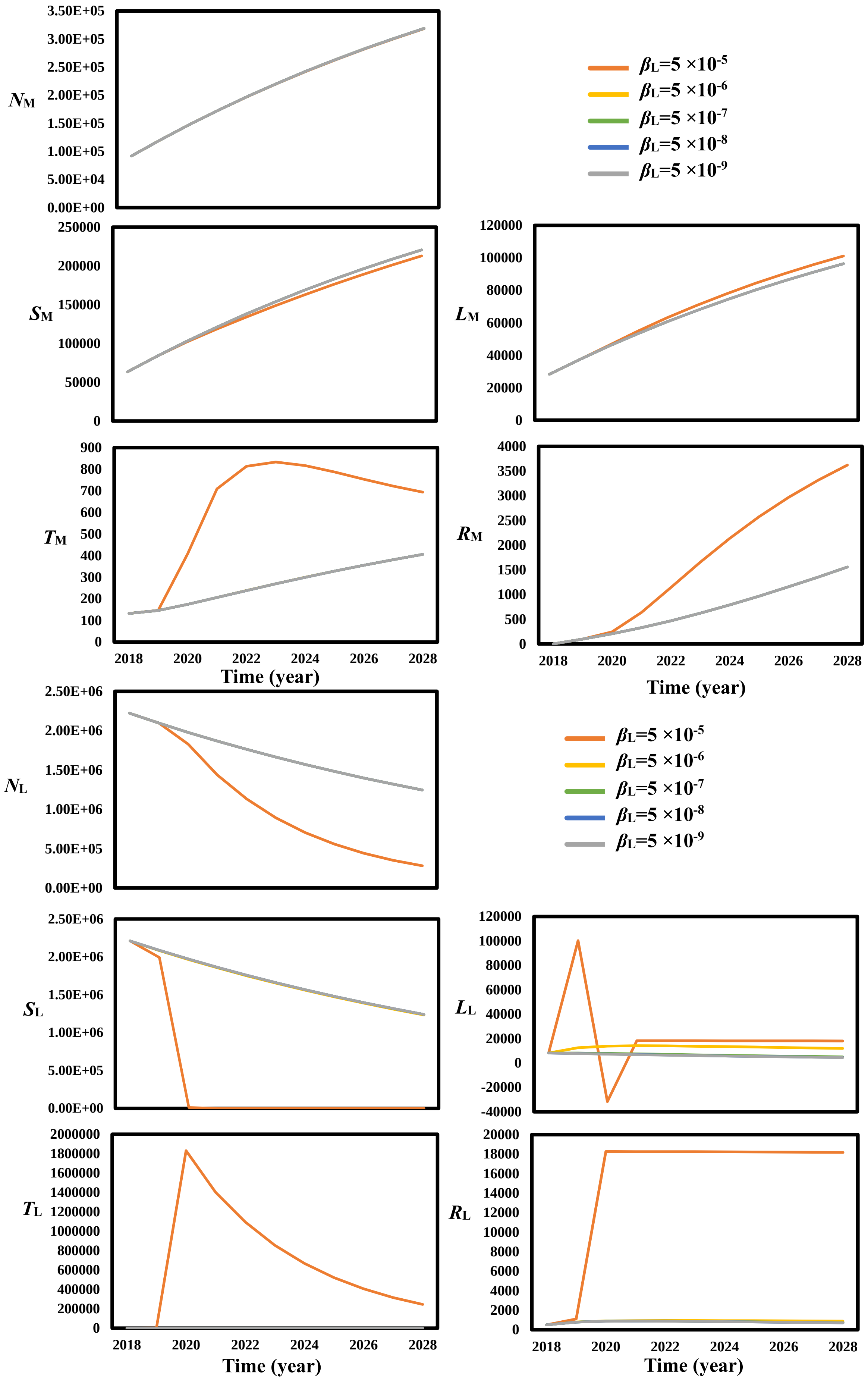
N_M
S_M
L_M
T_M
R_M
β_L=5 ×10^{-5}
β_L=5 ×10^{-6}
β_L=5 ×10^{-7}
β_L=5 ×10^{-8}
β_L=5 ×10^{-9}
N_L
S_L
L_L
T_L
R_L
2018
2020
2022
2024
2026
2028
Time (year)

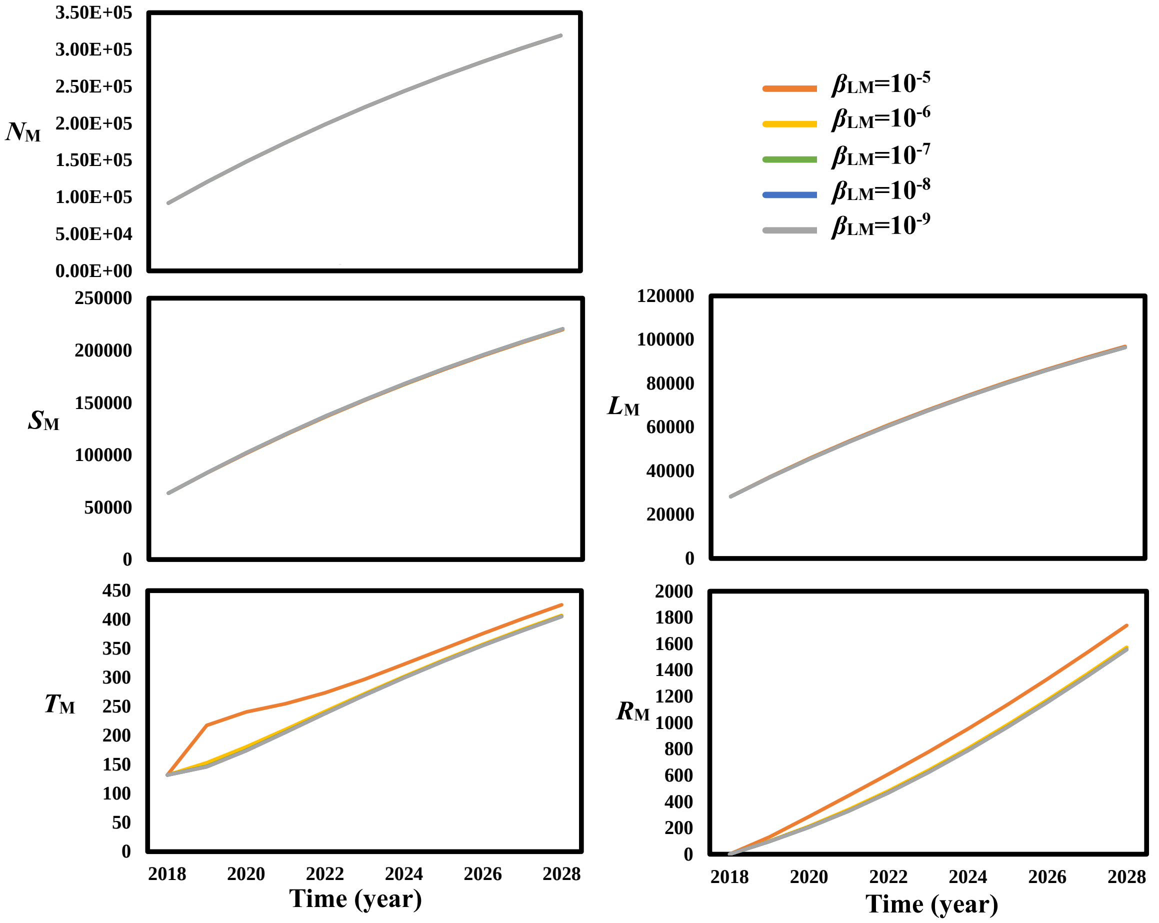

N_M
3.50E+05
3.00E+05
2.50E+05
2.00E+05
1.50E+05
1.00E+05
5.00E+04
0.00E+00
$\beta_{LM}=10^{-5}$
$\beta_{LM}=10^{-6}$
$\beta_{LM}=10^{-7}$
$\beta_{LM}=10^{-8}$
$\beta_{LM}=10^{-9}$
S_M
250000
200000
150000
100000
50000
0
L_M
120000
100000
80000
60000
40000
20000
0
T_M
450
400
350
300
250
200
150
100
50
0
R_M
2000
1800
1600
1400
1200
1000
800
600
400
200
0
2018
2020
2022
2024
2026
2028
Time (year)
Time (year)

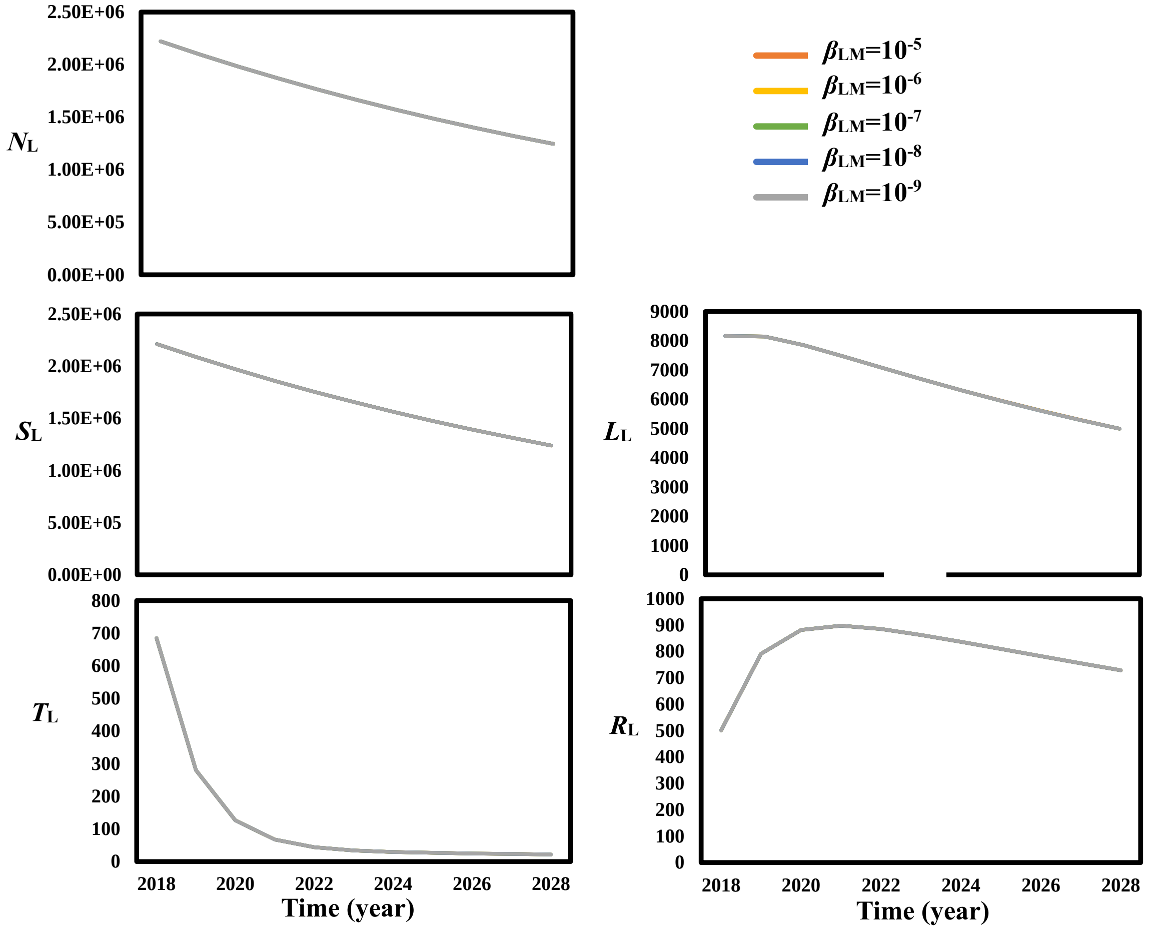

N_L
2.50E+06
2.00E+06
1.50E+06
1.00E+06
5.00E+05
0.00E+00
$\beta_{LM}=10^{-5}$
$\beta_{LM}=10^{-6}$
$\beta_{LM}=10^{-7}$
$\beta_{LM}=10^{-8}$
$\beta_{LM}=10^{-9}$
S_L
L_L
9000
8000
7000
6000
5000
4000
3000
2000
1000
0
T_L
800
700
600
500
400
300
200
100
0
R_L
1000
900
800
700
600
500
400
300
200
100
0
2018
2020
2022
2024
2026
2028
Time (year)
Time (year)

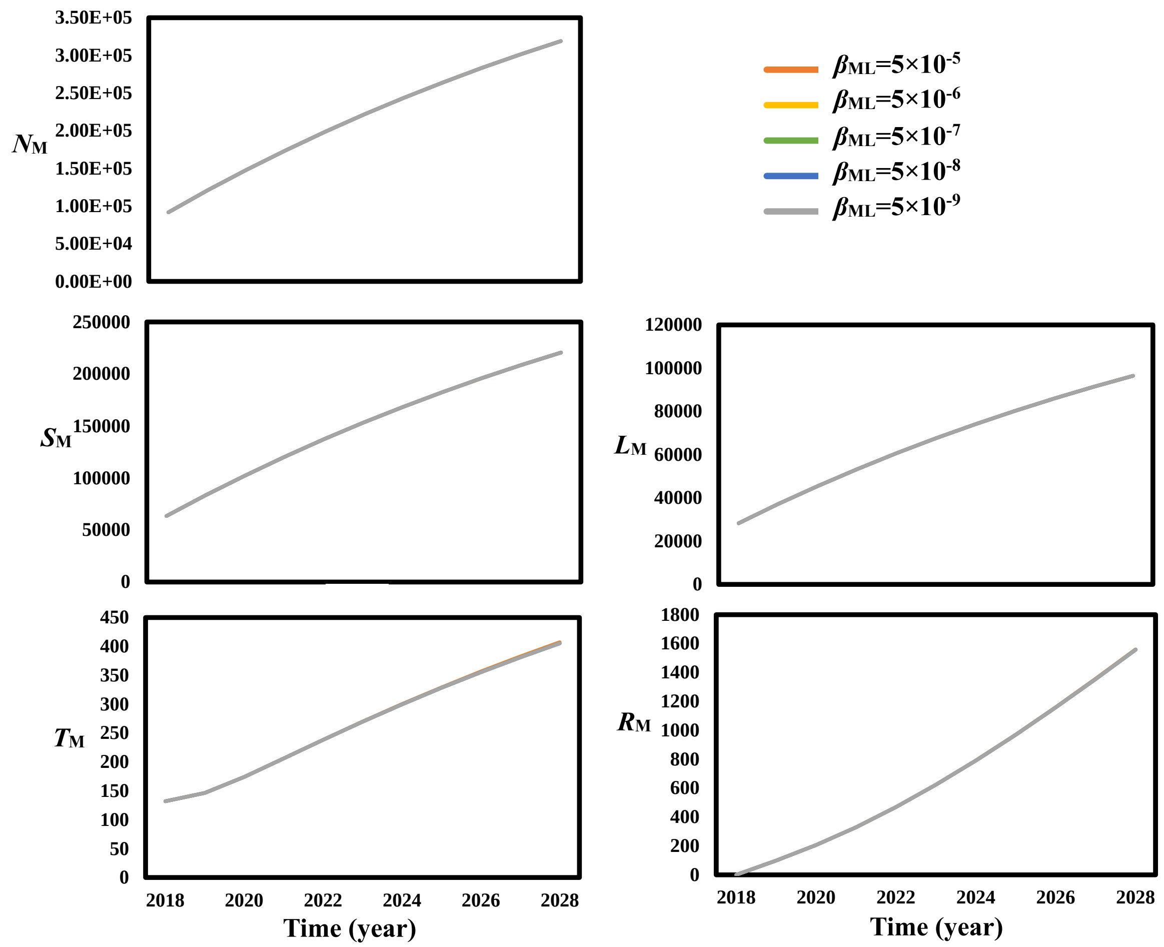

β_{ML}=5×10^{-5}
β_{ML}=5×10^{-6}
β_{ML}=5×10^{-7}
β_{ML}=5×10^{-8}
β_{ML}=5×10^{-9}
N_M
3.50E+05
3.00E+05
2.50E+05
2.00E+05
1.50E+05
1.00E+05
5.00E+04
0.00E+00
S_M
250000
200000
150000
100000
50000
0
L_M
120000
100000
80000
60000
40000
20000
0
T_M
450
400
350
300
250
200
150
100
50
0
R_M
1800
1600
1400
1200
1000
800
600
400
200
0
2018
2020
2022
2024
2026
2028
Time (year)
Time (year)

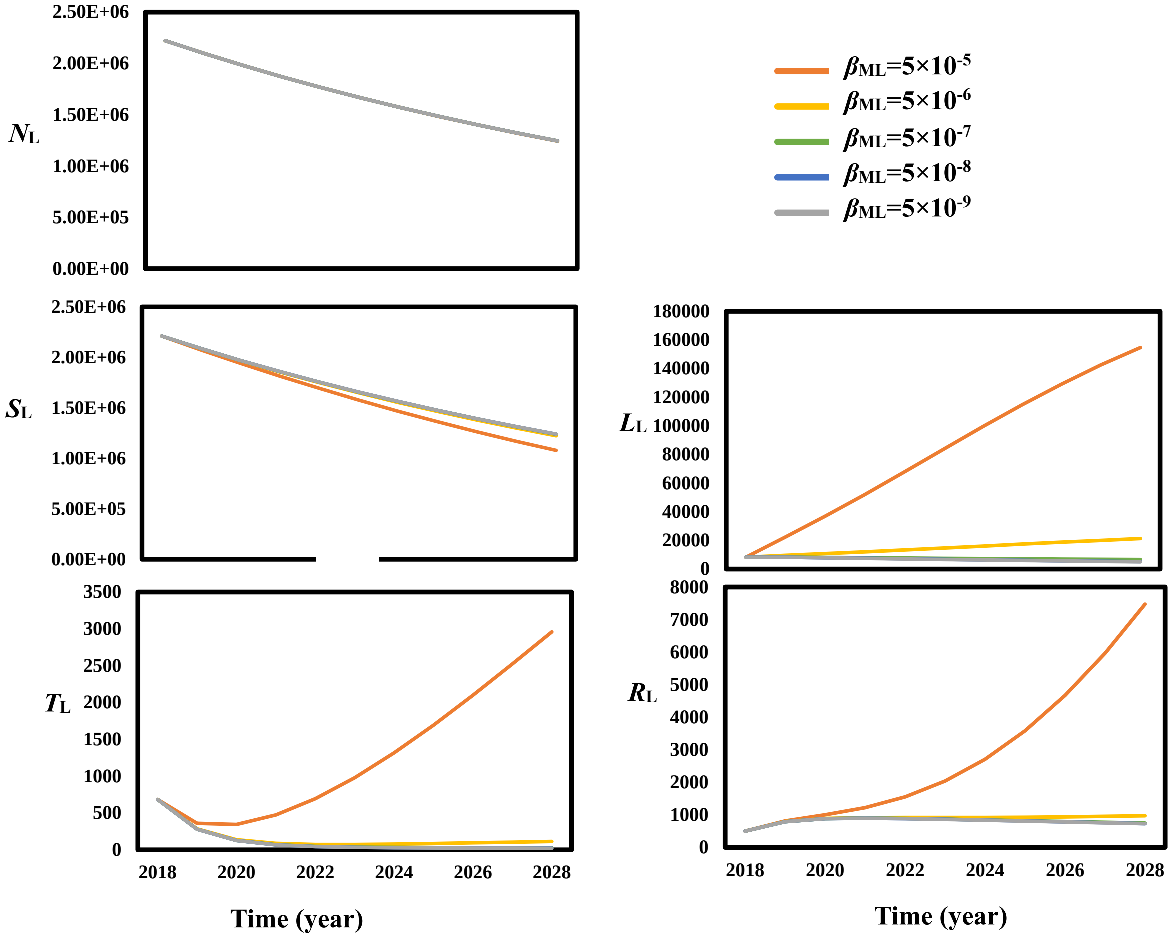

β_{ML}=5×10^{-5}
β_{ML}=5×10^{-6}
β_{ML}=5×10^{-7}
β_{ML}=5×10^{-8}
β_{ML}=5×10^{-9}
N_L
2.50E+06
2.00E+06
1.50E+06
1.00E+06
5.00E+05
0.00E+00
S_L
2.50E+06
2.00E+06
1.50E+06
1.00E+06
5.00E+05
0.00E+00
L_L
180000
160000
140000
120000
100000
80000
60000
40000
20000
0
T_L
3500
3000
2500
2000
1500
1000
500
0
R_L
8000
7000
6000
5000
4000
3000
2000
1000
0
2018
2020
2022
2024
2026
2028
Time (year)
Time (year)

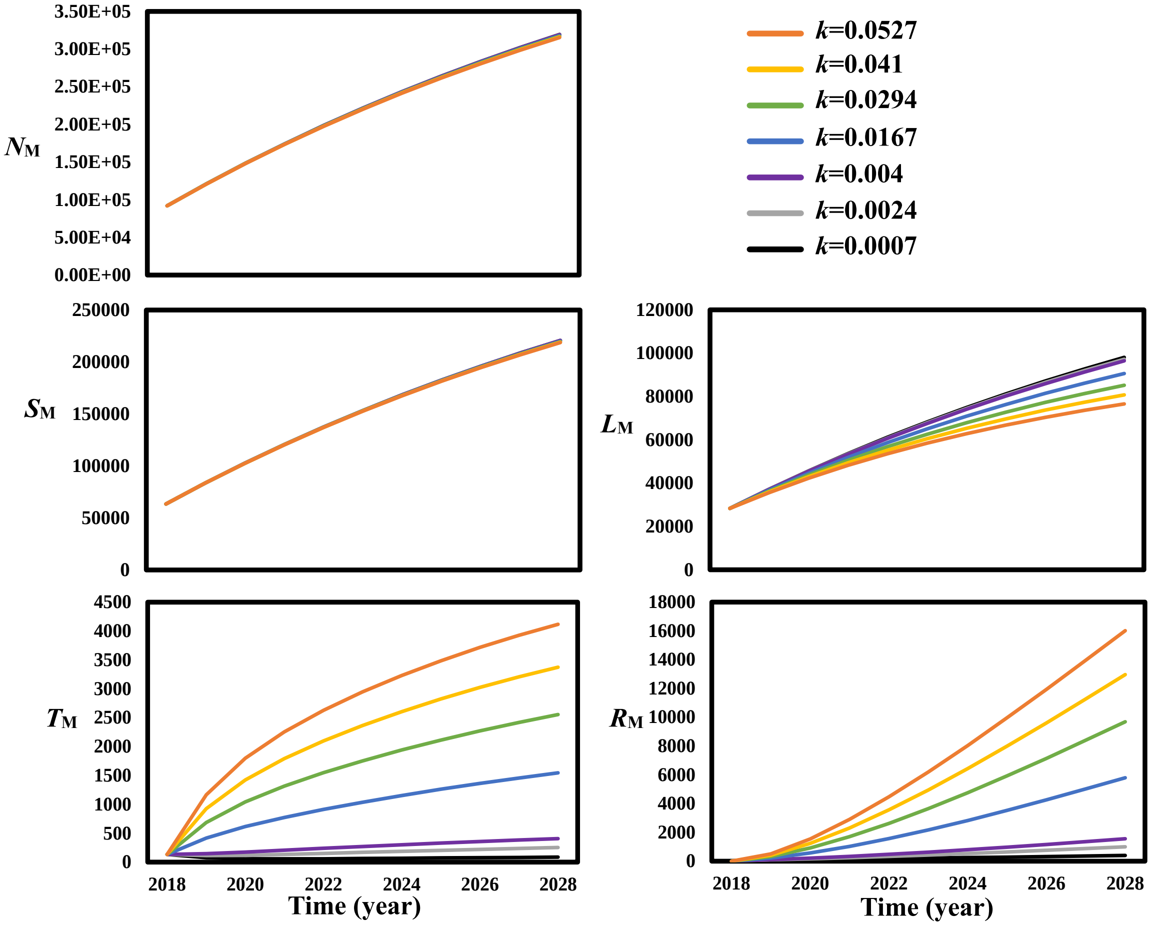
k=0.0527
k=0.041
k=0.0294
k=0.0167
k=0.004
k=0.0024
k=0.0007
NM
SM
LM
TM
RM
Time (year)
Time (year)

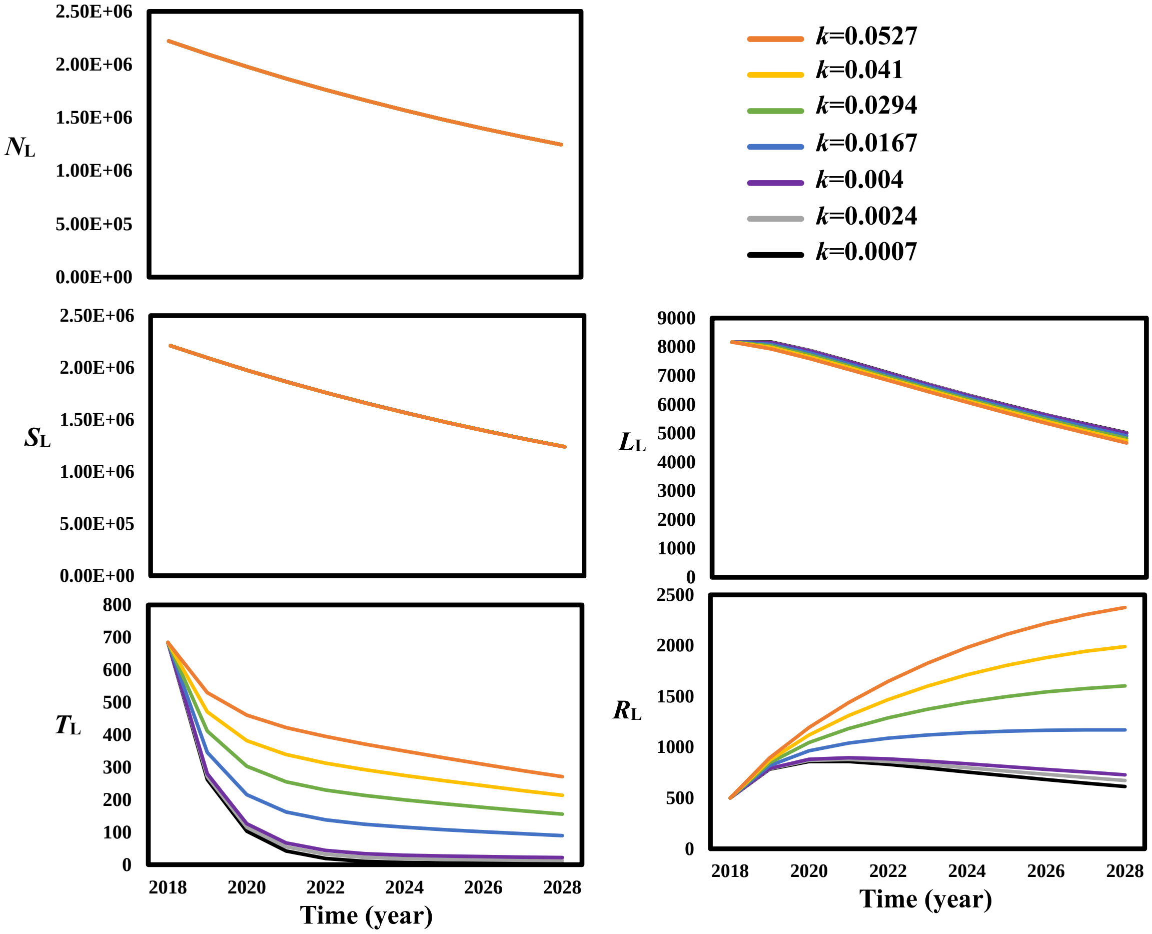
k=0.0527
k=0.041
k=0.0294
k=0.0167
k=0.004
k=0.0024
k=0.0007
NL
SL
LL
TL
RL
Time (year)
Time (year)

Figure S2 Sensitivity Analysis of four transmission rates (β_M, β_L, β_{LM}, β_{ML}) and reactivation rate (k) in Taichung City.

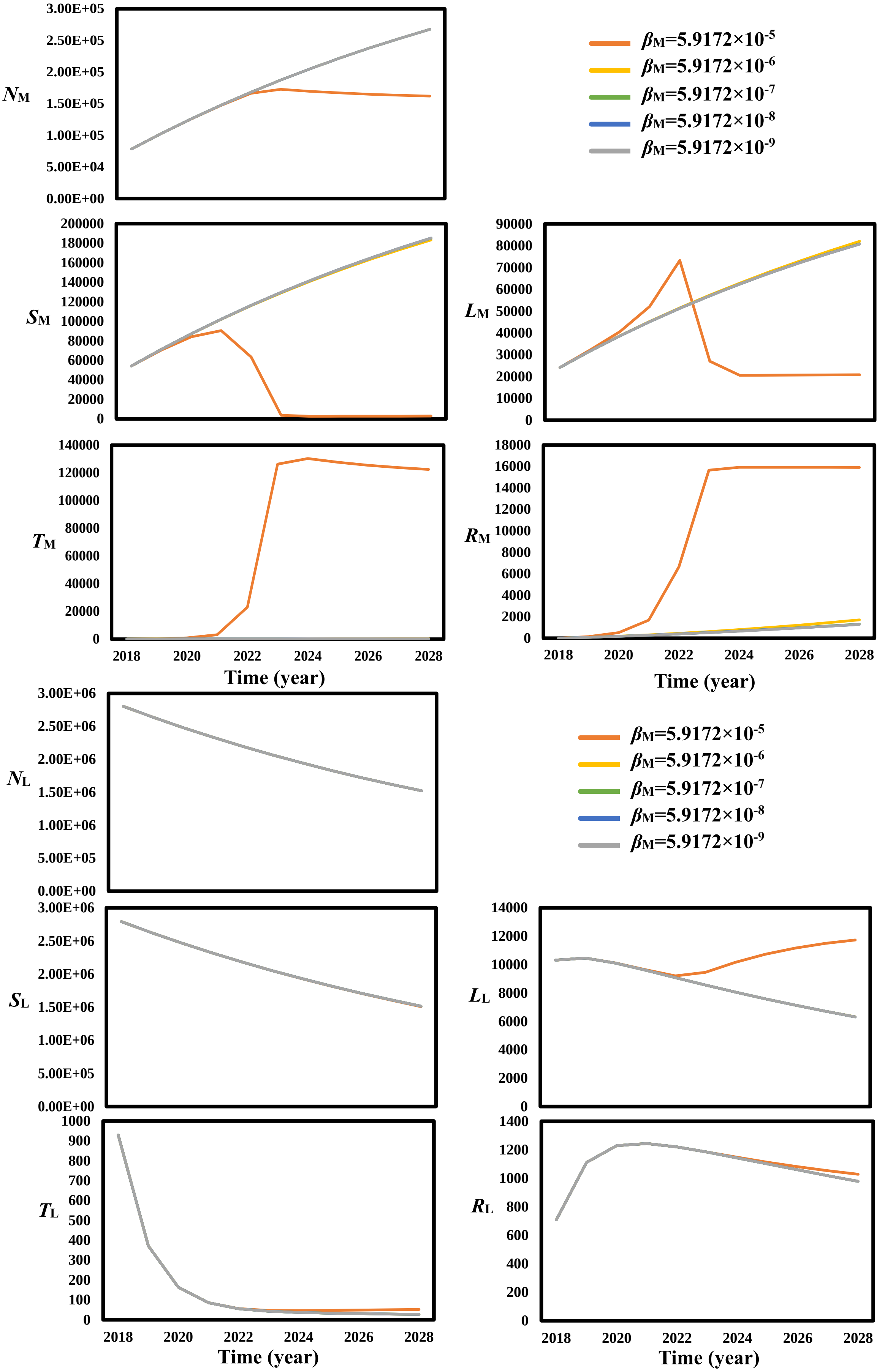

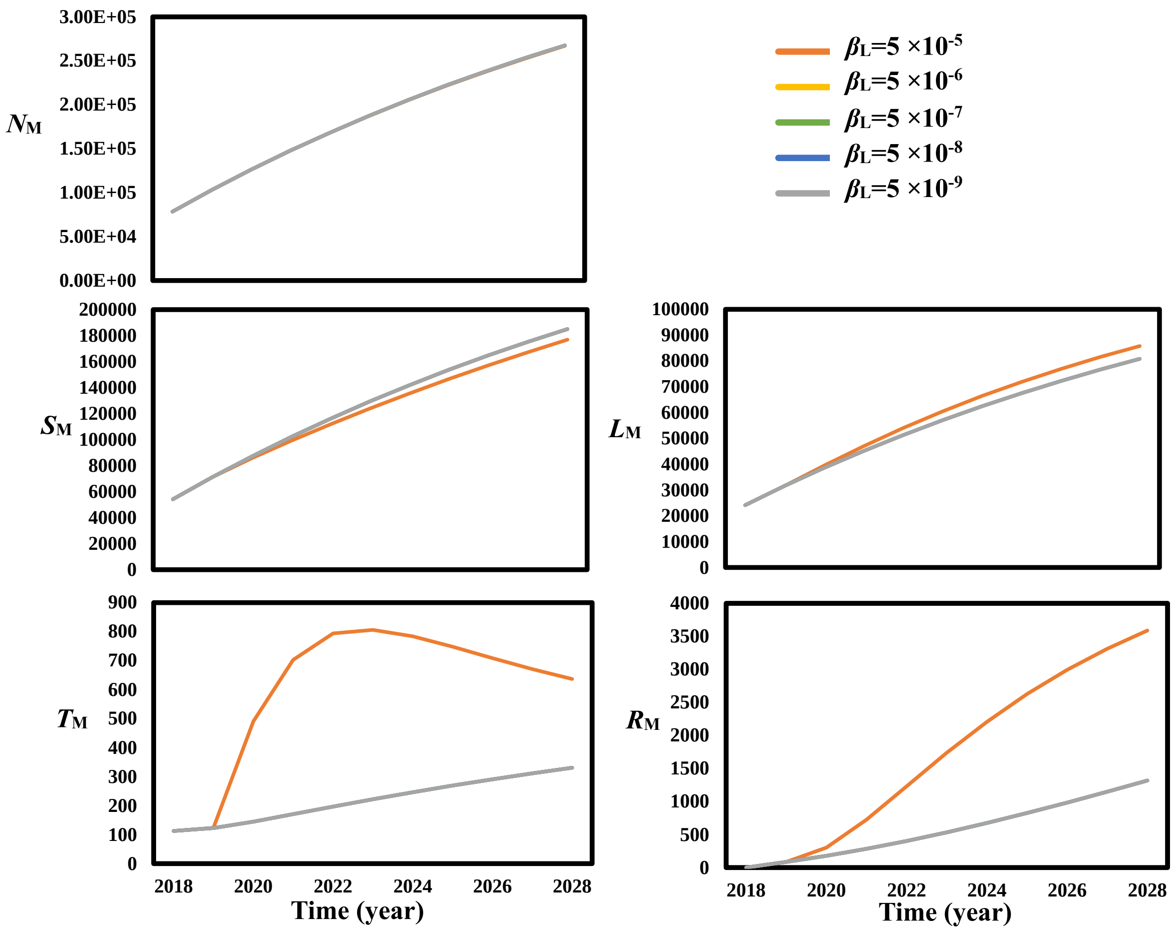

βL=5 ×10-5
βL=5 ×10-6
βL=5 ×10-7
βL=5 ×10-8
βL=5 ×10-9
NM
SM
LM
TM
RM
Time (year)
Time (year)

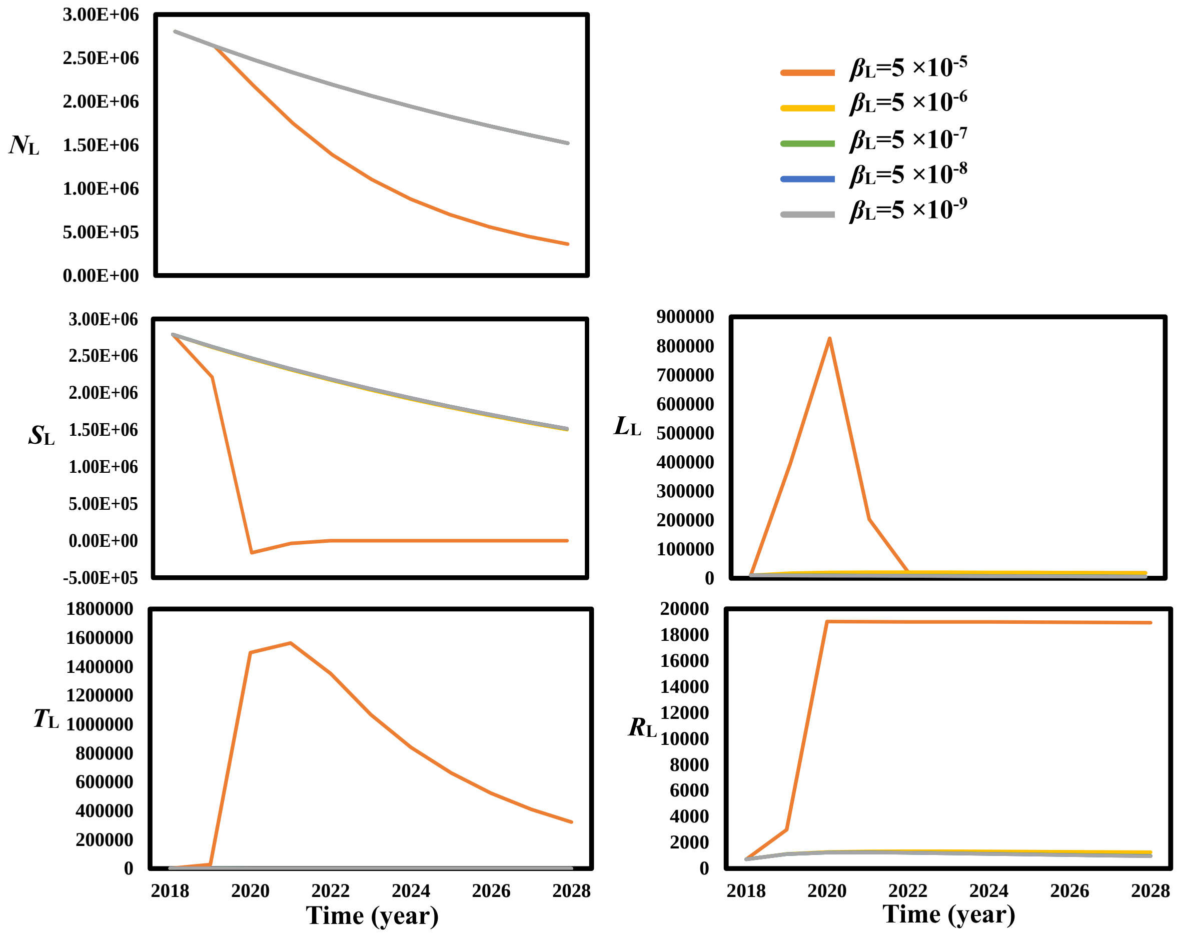

βL=5 ×10-5
βL=5 ×10-6
βL=5 ×10-7
βL=5 ×10-8
βL=5 ×10-9
NL
SL
LL
TL
RL
Time (year)
Time (year)

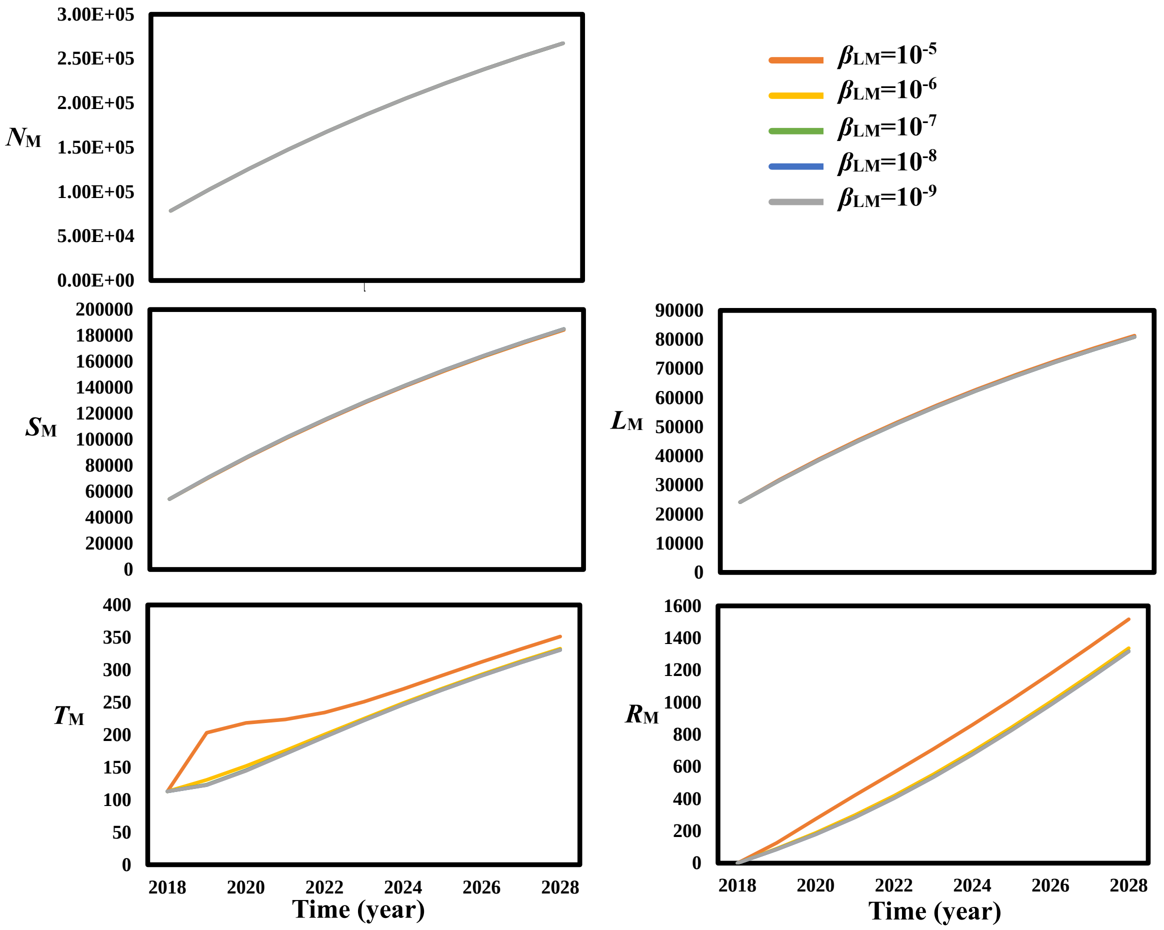

N_M
S_M
L_M
T_M
R_M
$\beta_{LM}=10^{-5}$
$\beta_{LM}=10^{-6}$
$\beta_{LM}=10^{-7}$
$\beta_{LM}=10^{-8}$
$\beta_{LM}=10^{-9}$
Time (year)
Time (year)

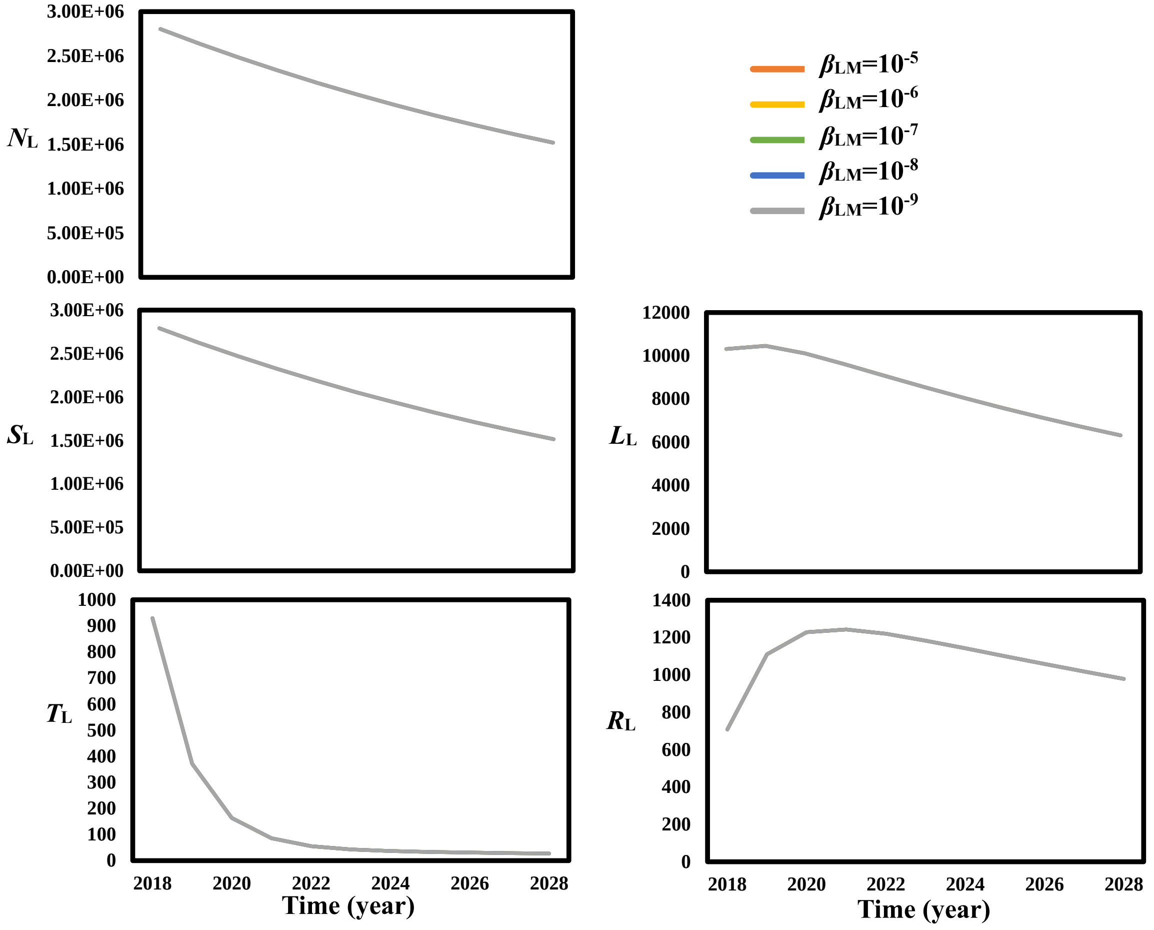

N_L
S_L
L_L
T_L
R_L
$\beta_{LM}=10^{-5}$
$\beta_{LM}=10^{-6}$
$\beta_{LM}=10^{-7}$
$\beta_{LM}=10^{-8}$
$\beta_{LM}=10^{-9}$
Time (year)
Time (year)

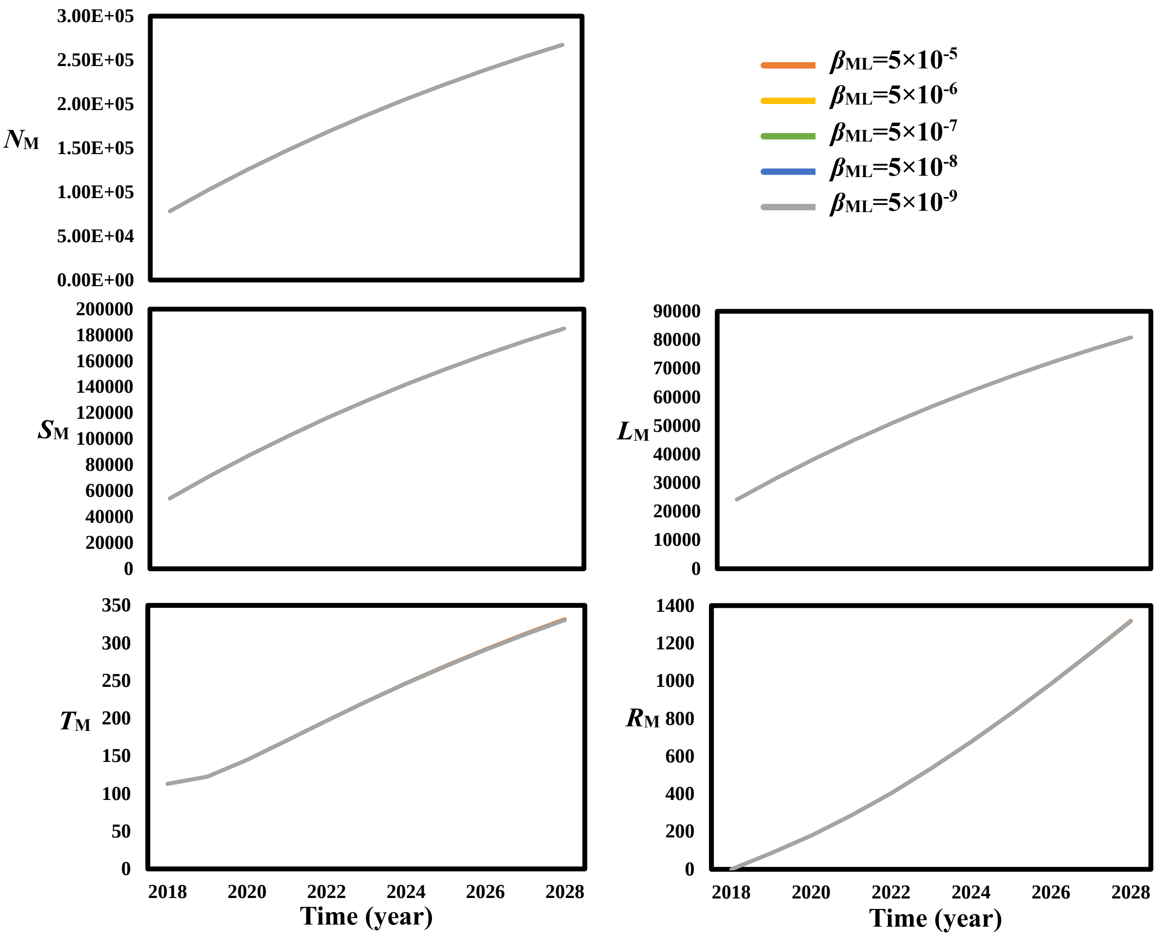

N_M
S_M
L_M
T_M
R_M
Time (year)
$\beta_{ML}=5\times10^{-5}$
$\beta_{ML}=5\times10^{-6}$
$\beta_{ML}=5\times10^{-7}$
$\beta_{ML}=5\times10^{-8}$
$\beta_{ML}=5\times10^{-9}$

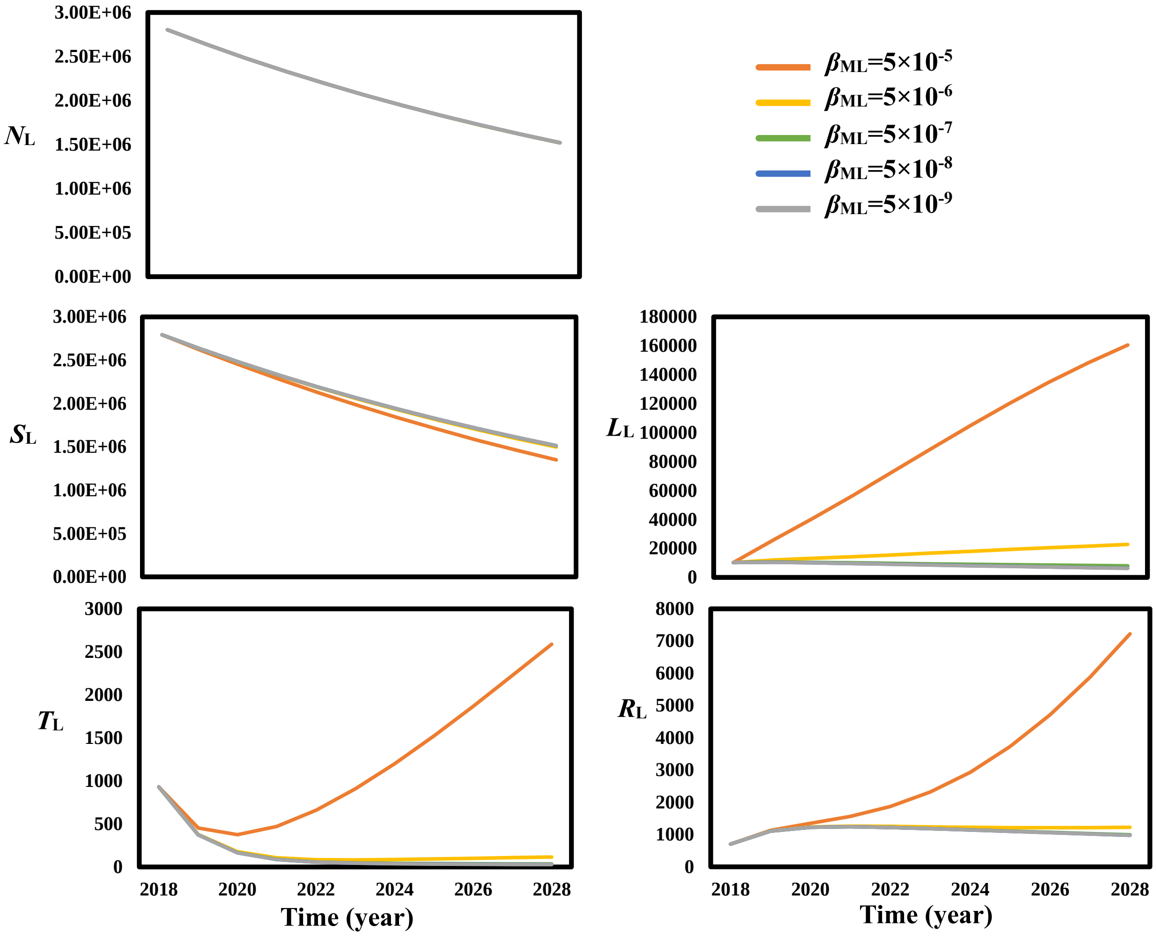

N_L
S_L
L_L
T_L
R_L
Time (year)
$\beta_{ML}=5\times10^{-5}$
$\beta_{ML}=5\times10^{-6}$
$\beta_{ML}=5\times10^{-7}$
$\beta_{ML}=5\times10^{-8}$
$\beta_{ML}=5\times10^{-9}$

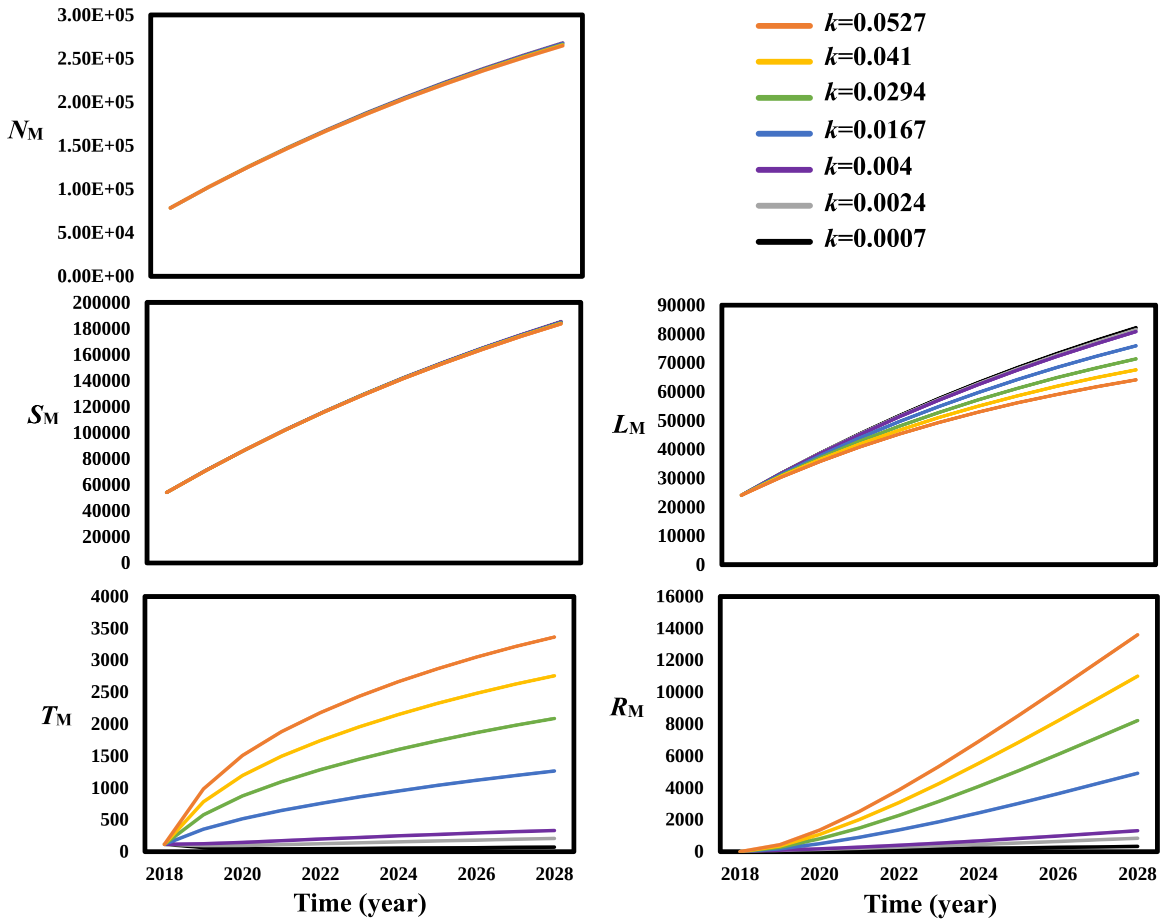

k=0.0527
k=0.041
k=0.0294
k=0.0167
k=0.004
k=0.0024
k=0.0007
NM
3.00E+05
2.50E+05
2.00E+05
1.50E+05
1.00E+05
5.00E+04
0.00E+00
SM
200000
180000
160000
140000
120000
100000
80000
60000
40000
20000
0
LM
90000
80000
70000
60000
50000
40000
30000
20000
10000
0
TM
4000
3500
3000
2500
2000
1500
1000
500
0
RM
16000
14000
12000
10000
8000
6000
4000
2000
0
2018
2020
2022
2024
2026
2028
Time (year)
Time (year)

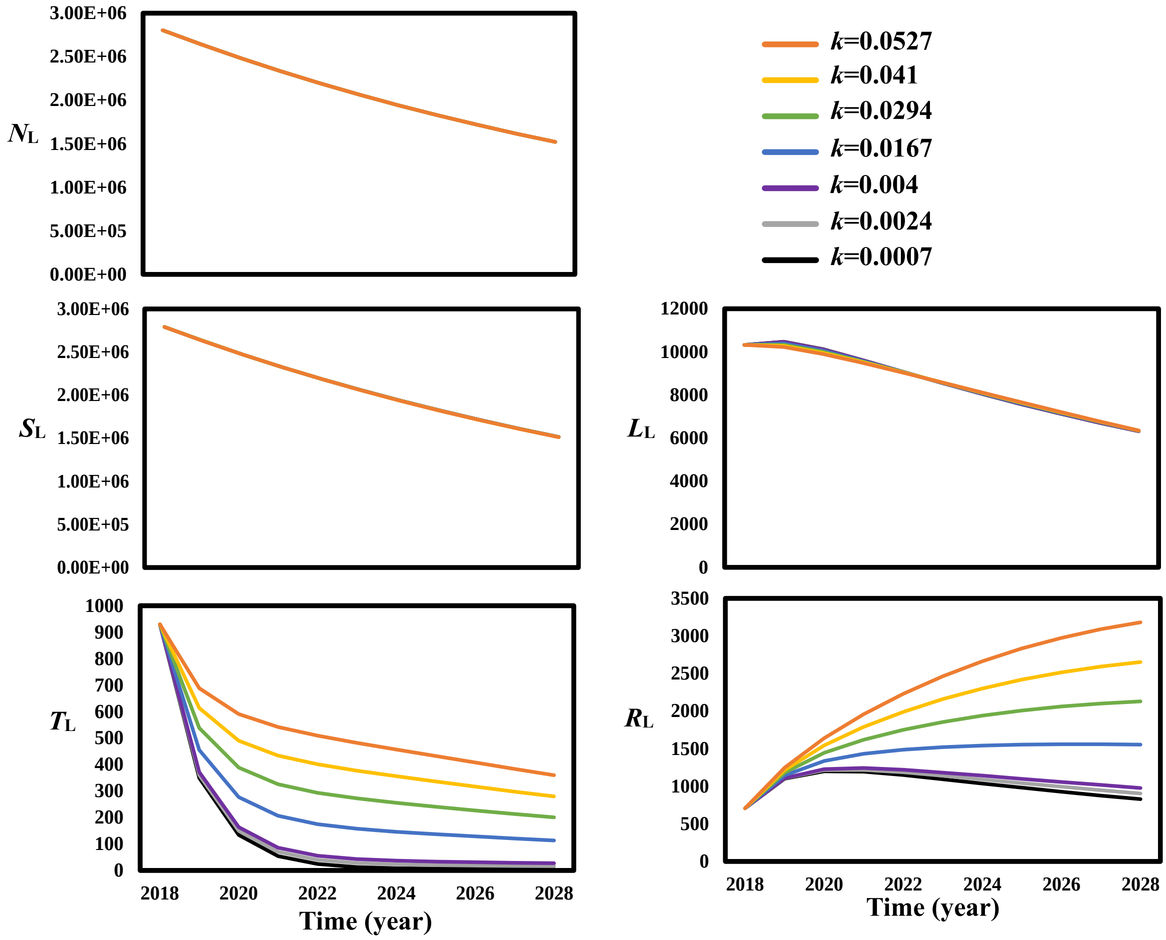

k=0.0527
k=0.041
k=0.0294
k=0.0167
k=0.004
k=0.0024
k=0.0007
NL
3.00E+06
2.50E+06
2.00E+06
1.50E+06
1.00E+06
5.00E+05
0.00E+00
SL
3.00E+06
2.50E+06
2.00E+06
1.50E+06
1.00E+06
5.00E+05
0.00E+00
LL
12000
10000
8000
6000
4000
2000
0
TL
1000
900
800
700
600
500
400
300
200
100
0
RL
3500
3000
2500
2000
1500
1000
500
0
2018
2020
2022
2024
2026
2028
Time (year)
Time (year)

Figure S3 Sensitivity Analysis of four transmission rates (β_M, β_L, β_{LM}, β_{ML}) and reactivation rate (k) in New Taipei City.

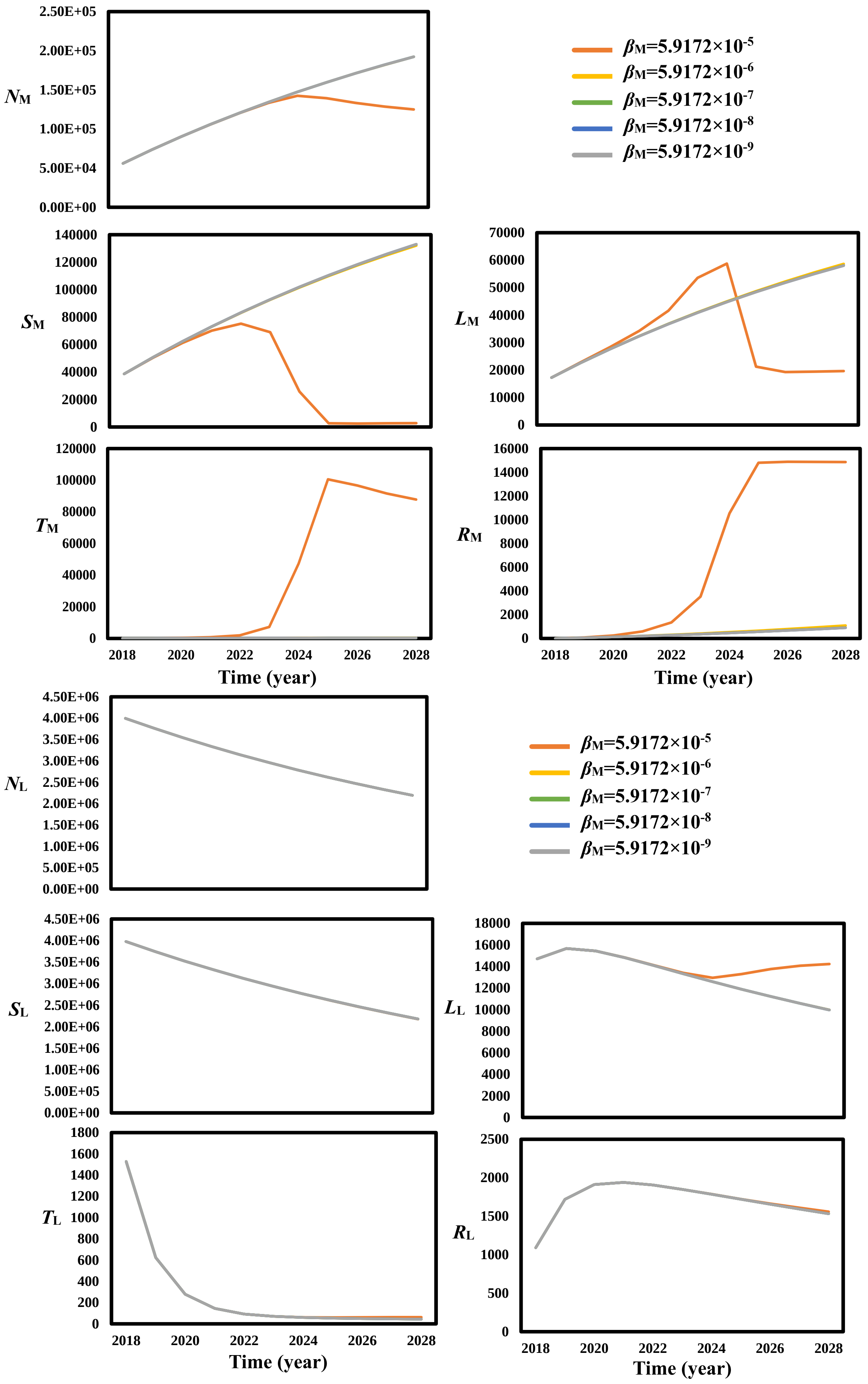

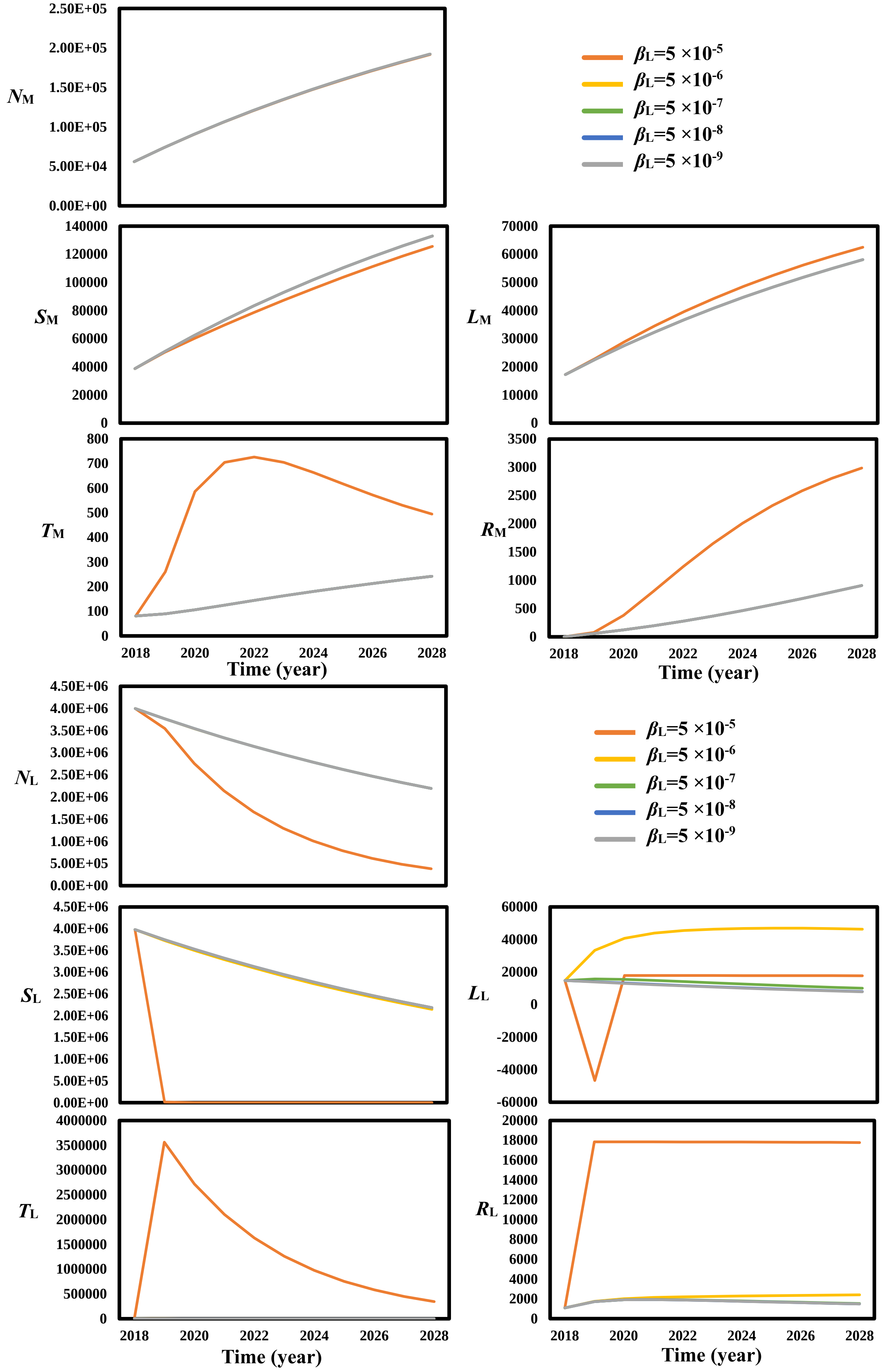

β_L=5 ×10^{-5}
β_L=5 ×10^{-6}
β_L=5 ×10^{-7}
β_L=5 ×10^{-8}
β_L=5 ×10^{-9}
N_M
S_M
L_M
T_M
R_M
N_L
S_L
L_L
T_L
R_L
2018
2020
2022
2024
2026
2028
Time (year)

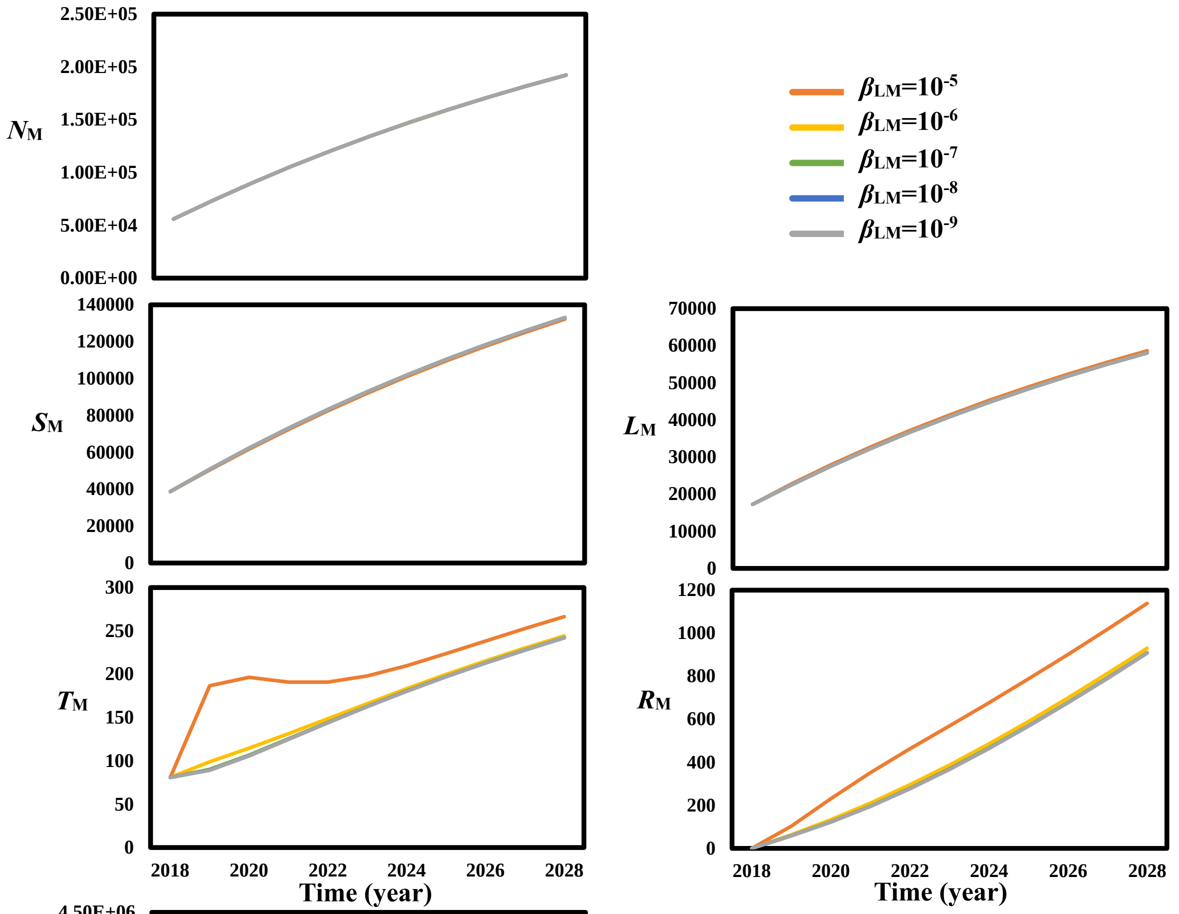
$\beta_{LM}=10^{-5}$
$\beta_{LM}=10^{-6}$
$\beta_{LM}=10^{-7}$
$\beta_{LM}=10^{-8}$
$\beta_{LM}=10^{-9}$
N_M
S_M
L_M
T_M
R_M
2018
2020
2022
2024
2026
2028
Time (year)

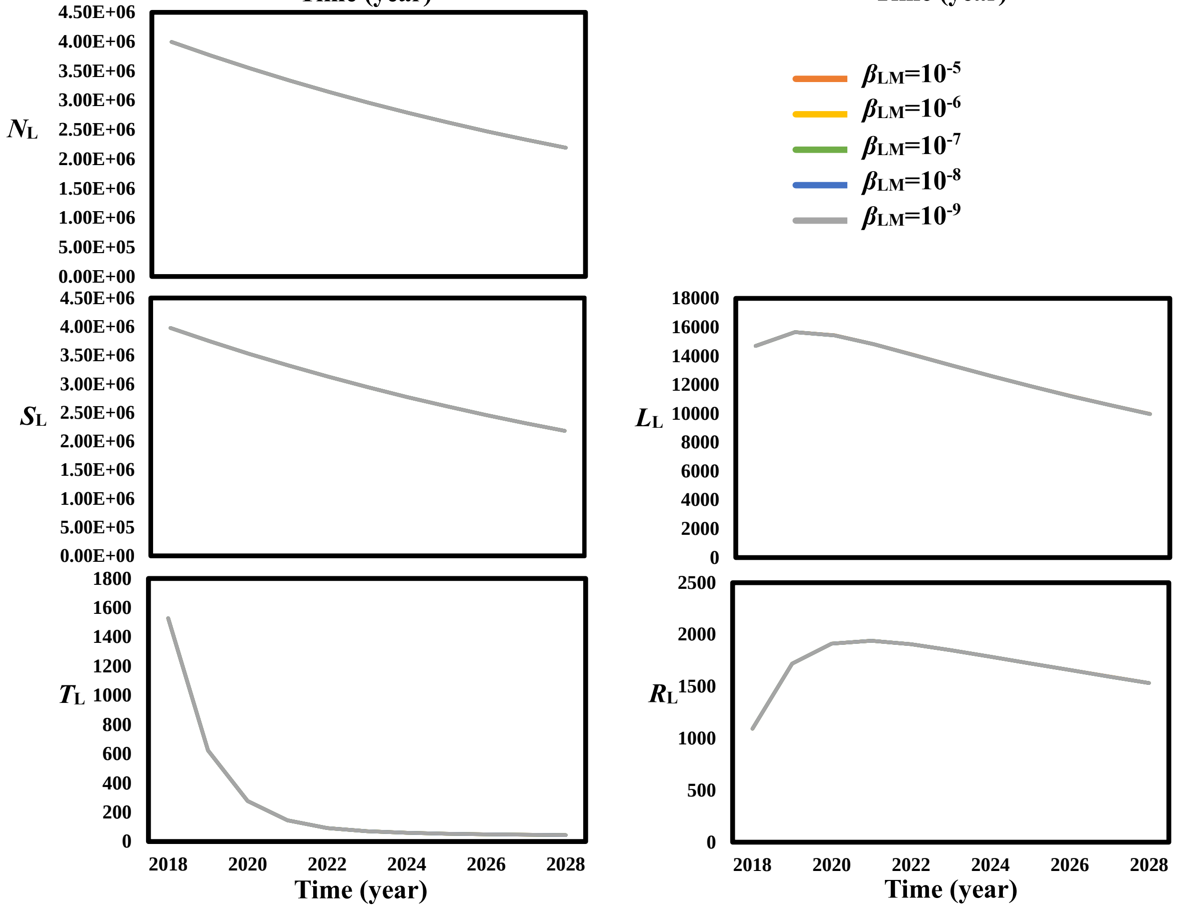
$\beta_{LM}=10^{-5}$
$\beta_{LM}=10^{-6}$
$\beta_{LM}=10^{-7}$
$\beta_{LM}=10^{-8}$
$\beta_{LM}=10^{-9}$
N_L
S_L
L_L
T_L
R_L
2018
2020
2022
2024
2026
2028
Time (year)

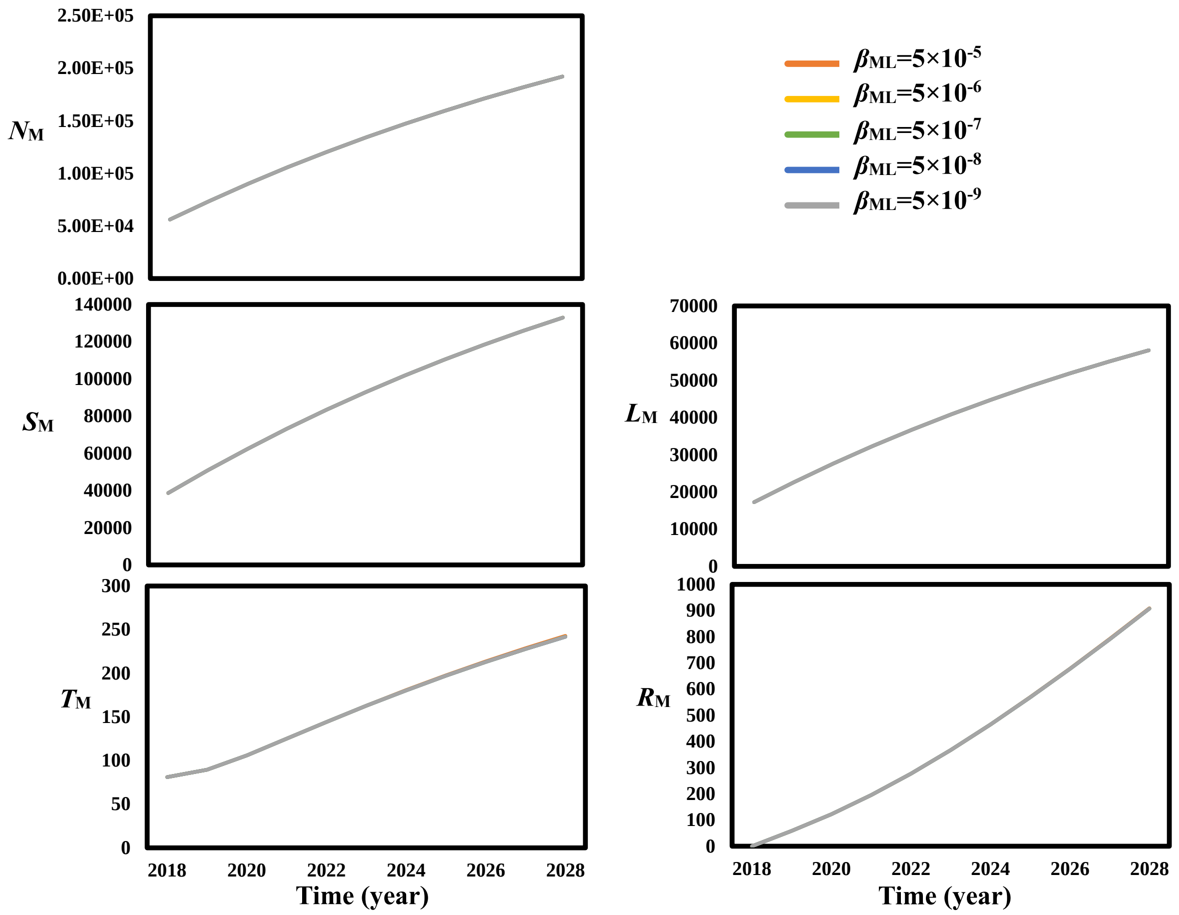

N_M
S_M
L_M
T_M
R_M
β_{ML}=5×10^{-5}
β_{ML}=5×10^{-6}
β_{ML}=5×10^{-7}
β_{ML}=5×10^{-8}
β_{ML}=5×10^{-9}
2018
2020
2022
2024
2026
2028
Time (year)
Time (year)

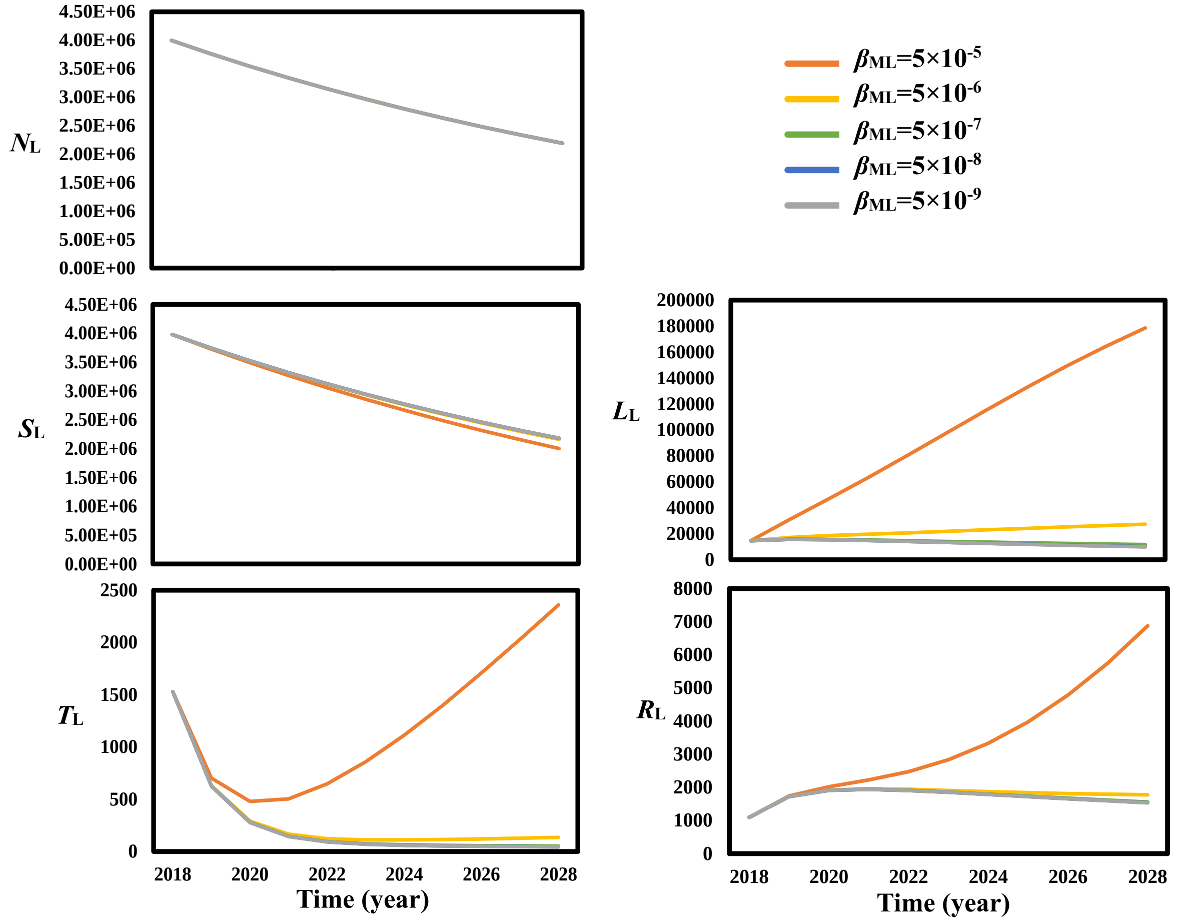

N_L
S_L
L_L
T_L
R_L
β_{ML}=5×10^{-5}
β_{ML}=5×10^{-6}
β_{ML}=5×10^{-7}
β_{ML}=5×10^{-8}
β_{ML}=5×10^{-9}
2018
2020
2022
2024
2026
2028
Time (year)
Time (year)

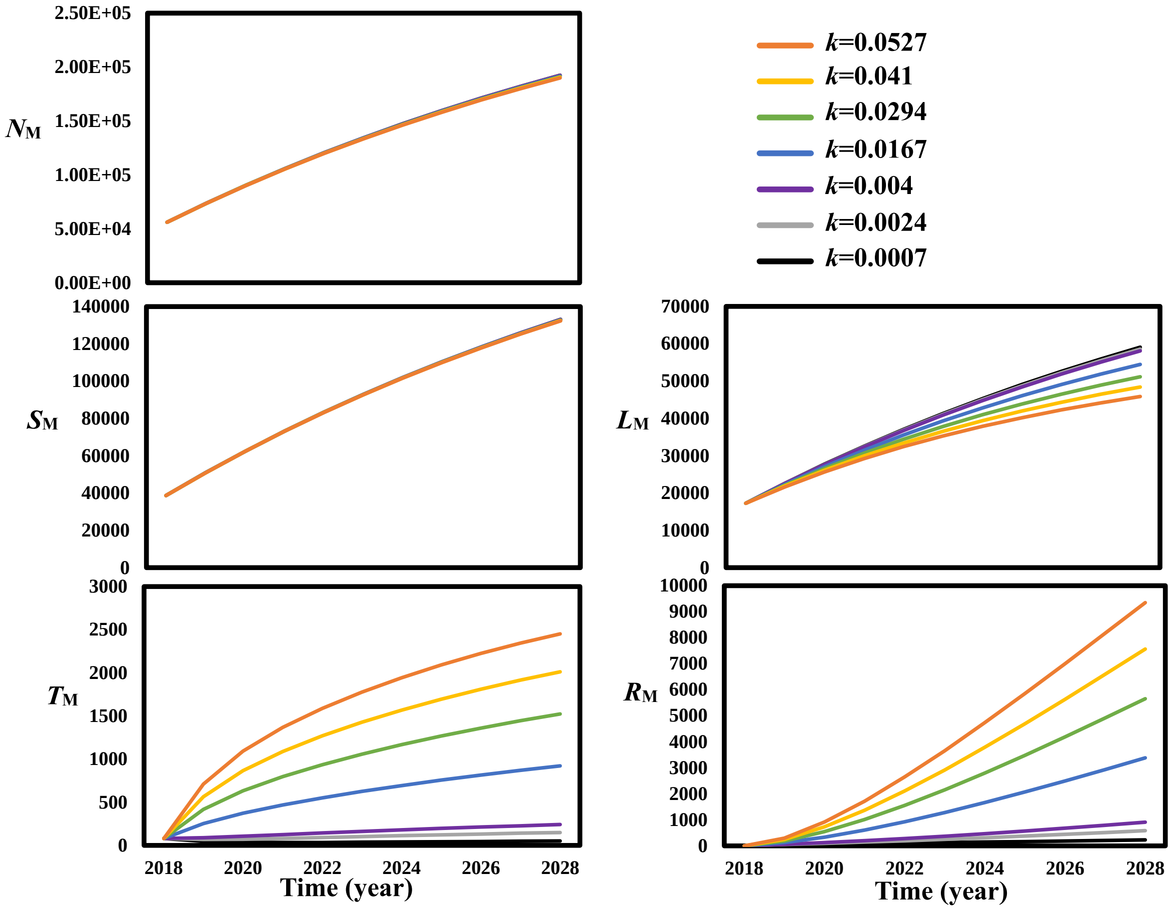

NM
SM
LM
TM
RM
k=0.0527
k=0.041
k=0.0294
k=0.0167
k=0.004
k=0.0024
k=0.0007
Time (year)
Time (year)

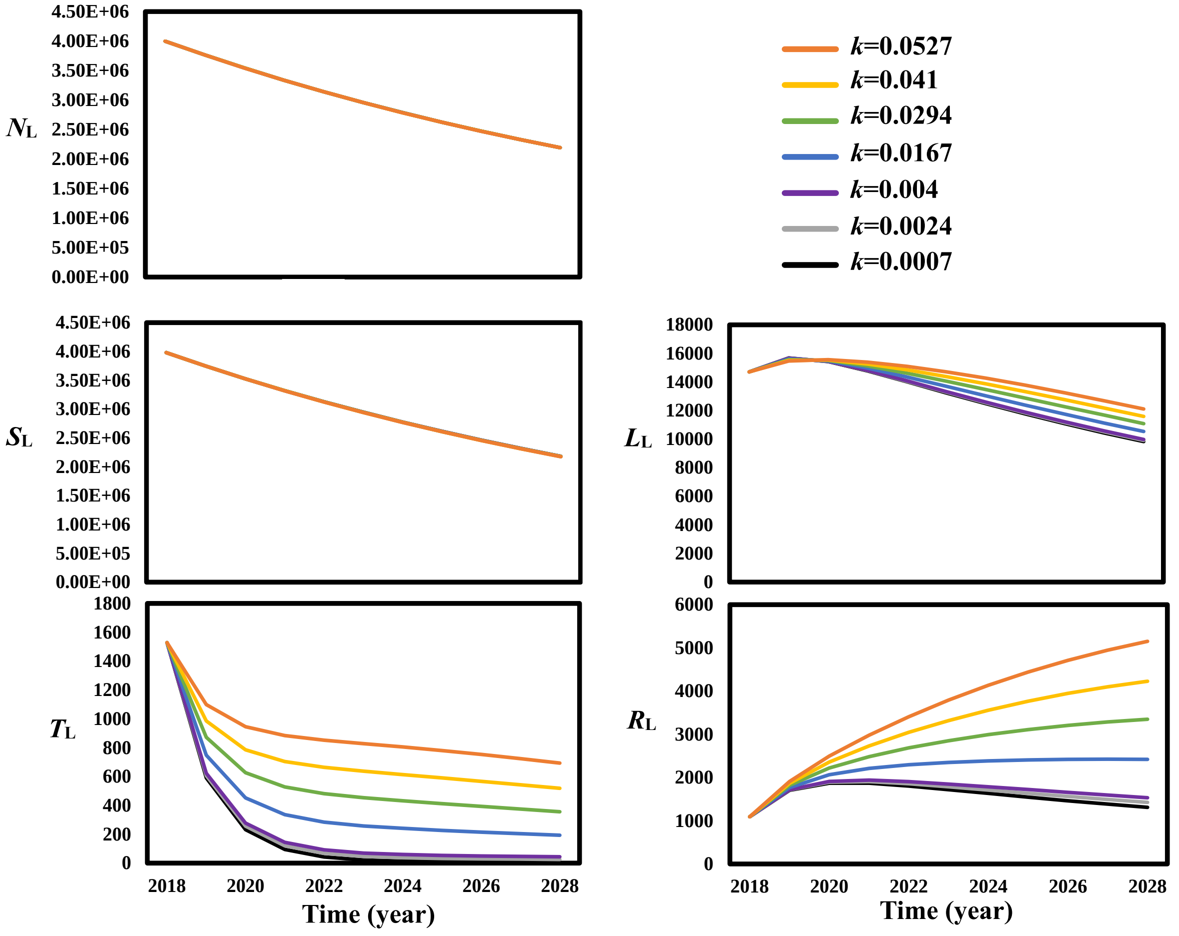

NL
SL
LL
TL
RL
k=0.0527
k=0.041
k=0.0294
k=0.0167
k=0.004
k=0.0024
k=0.0007
Time (year)
Time (year)
